# Mining laboratory data to describe prostate specific antigen testing and prostate cancer in Johannesburg, South Africa

**DOI:** 10.11604/pamj.2020.35.61.21331

**Published:** 2020-02-28

**Authors:** Mpho Reginah Maphayi, Naseem Cassim, Braimoh Bello, Jaya Anna George

**Affiliations:** 1 Department of Chemical Pathology, University of the Witwatersrand and National Health Laboratory Service, Johannesburg, South Africa; 2 Department of Molecular Medicine and Hematology, University of the Witwatersrand and National Health Laboratory Service, Johannesburg, South Africa; 3 School of Public Health, Faculty of Health Sciences, University of Witwatersrand, Johannesburg, South Africa

**Keywords:** Screening, prostate specific antigen, prostate biopsy, racial disparity

## Abstract

**Introduction:**

guidelines issued by different organizations worldwide differ on the use of prostate specific antigen (PSA) in prostate cancer. However, no local data is available describing how PSA testing is offered by our healthcare facilities in the country. The objectives of this study were to describe PSA testing and subsequent prostate biopsy uptake in a South African urban population.

**Methods:**

this was a descriptive retrospective study. Data of all PSA tests and prostate biopsies performed at Charlotte Maxeke Johannesburg Academic Hospital (CMJAH) laboratory for 2013 calendar year was extracted from the laboratory information system.

**Results:**

a total of 20 365 PSA tests were performed on 17 481 men during the study period. The majority of men were Black African (79%). The mean age for Black Africans (55.5 years, SD 13.3) was significantly lower than other racial groups (62.9 years, SD 12.6, p < 0.0005). PSA level was lower in Black Africans compared to others. Prostate biopsy uptake across all age groups was lower in Black African men compared to others (2% versus 4%, p = 0.01). Of the 423 men who had a prostate biopsy, 50% had prostate cancer. More Black African men were diagnosed with prostate cancer on biopsy compared to men of other racial groups (54% versus 43%, p = 0.03).

**Conclusion:**

our study confirms that PSA testing is prevalent in healthcare facilities in South Africa. Black African men are tested for PSA levels but have low biopsy uptake and are more likely to be diagnosed with prostate cancer.

## Introduction

Prostate cancer is the second most common type of cancer affecting men in the world and the fifth leading cause of cancer death [1]. Prostate cancer incidence is highest in developed countries such as Australia, New Zealand and North America. Countries with predominantly black populations such as the Caribbean and sub-Saharan Africa have the highest mortality rates [1]. Generally, prostate cancer incidence is low for Africans when compared with African American men. Nonetheless, this is expected to increase with improvement in life expectancy, access to healthcare and screening facilities in African countries [2]. A meta-analysis by Adeloye *et al.* in 2016 reported an estimated pooled incidence of 22.0 (95% confidence interval of 19.3-23.0) per 100 000 men across the African continent, with the highest incidences in sub-Saharan Africa [3]. However, this may be underestimated as many African countries do not have functional, up-to-date national cancer registries [3]. Furthermore, only 40 studies from 16 African countries were eligible for inclusion in the meta-analysis and the majority were from Western and Southern Africa [3]. In South Africa, there seem to be racial disparities in prostate cancer. Both prostate cancer incidence and mortality rates are highest among Black African men [4, 5].

One of the risk factors for developing prostate cancer is African ancestry [6]. African American men are at higher risk of developing prostate cancer than their white counterparts [6]. Similar trends have been observed in South Africa. Black South African men are not only at higher risk of developing aggressive prostate cancer but also tend to present late [7, 8], and the few that present at younger age (40-50 years or younger) with prostate cancer, have poor prognosis [9]. One of the factors contributing to racial disparity in prostate cancer presentation amongst South African men is limited access to screening and early detection facilities [7, 10]. Prostate specific antigen (PSA), a serine protease produced by the prostate gland that has been used in prostate cancer diagnosis and management for decades. However, it is not prostate cancer specific as it is also increased after prostate gland manipulation (digital rectal examination, transurethral ultrasound), urinary tract infection and benign conditions of the prostate such as benign prostate hypertrophy and prostatitis [11]. Despite its limitations, PSA testing has improved the early detection of prostate cancer in most countries [12]. The increase in use of PSA observed in countries with high incidences of prostate cancer has enabled early detection of organ confined cancer and early interventions [1, 13, 14]. It has also been established that PSA based screening reduces the prostate cancer mortality rate in men between 55 and 69 years old [12, 15]. One of the risk of PSA testing is diagnosing and treating latent cancer that would not have clinically manifested [12, 15, 16]. Despite this, guidelines on screening for prostate cancer using PSA differ amongst the various medical societies [17, 18].

In South Africa, efforts to improve early diagnosis will include PSA testing. The local guidelines issued by the Prostate Cancer Foundation of South Africa recommend PSA testing for Black African men and men with family history of prostate cancer and/or breast cancer from age 40 years, all other men from age 45 and other men with lower urinary tract or other symptoms suggestive of prostate cancer [11]. The foundation further recommends using age-specific total PSA reference ranges. Unfortunately, these ranges have not been validated for our population. Furthermore, the National Health Laboratory Services (NHLS), comprising the nation’s public health laboratories report PSA results using a fixed cut-off of 4.00 µg/L. The aim of our study was to understand how PSA testing in South African men can be used to improve early and proper diagnosis of prostate cancer. The specific objectives were to: describe PSA testing performed at the NHLS laboratory and compare PSA levels between Black African men and men of other racial groups. The results are important for a country with one of the highest prostate cancer mortality rate in the world and for Black African men in general [1].

## Methods

**Study design and setting:** this was a descriptive retrospective study using data from the NHLS Laboratory Information System (LIS). The study sample included all PSA tests done at the Charlotte Maxeke Johannesburg Academic Hospital (CMJAH) NHLS laboratory in the 2013 calendar year. This laboratory serves both the local hospital and surrounding healthcare facilities. It receives test requests from over 100 public healthcare facilities from within and outside the Gauteng province. It is situated in the Johannesburg metropolitan area in Gauteng province, South Africa. Johannesburg is not only the economic hub of South Africa but also the most populated area of the country [19].

**Figure 1:**
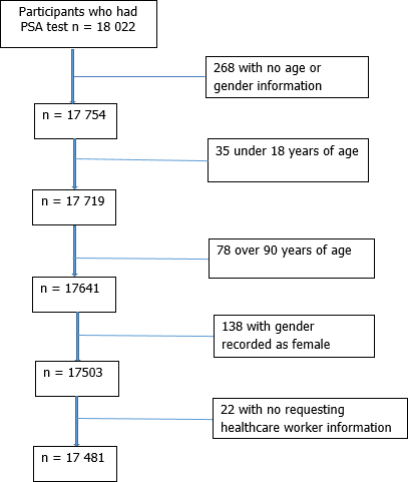
The number of participants included in the analysis after applying study exclusion criteria

**Study population and exclusion criteria:** study participants included men for whom a PSA test was performed at the CMJAH laboratory. We excluded cases where information of the requesting healthcare worker (HCW), health care facility and patient demographics such as gender and age were not provided (Figure 1). Prostate specific antigen tests for cases for which the record gender was not male were also excluded in the analysis because PSA testing is only recommended in males [20].

**Laboratory measurement of prostate specific antigen:** total PSA in serum was measured by two-site sandwich immunoassay, using chemiluminometric technology (Advia Centuar) from Siemens (Munich, Germany). The measuring range for the assay is 0.01-100 µg/L [20]. Results above this range were analysed in dilution according to the manufacturer’s recommendation [20]. Across the NHLS, laboratories report PSA results using a cut-off of < 4.00 µg/L. Results above this cut-off are considered high and requiring further investigations.

**Other variables:** we categorized healthcare facilities as either hospital or clinics. For study participants, only the first five results were included. Data analysis for the first PSA result performed during the study period was done for all participants. Subsequent PSA results were used to assess the extent of repeat testing. The requesting healthcare workers were categorised as doctors or nurses using their profession regulatory body registration number. Study participants’ races were categorised as Black African and Other. Information on other racial groups was not available from data retrieved from LIS. The participants’ age was required for categorising their PSA results according to age specific reference intervals as per the local guidelines [11]. They were divided into four categories: less than 40 years’ old, 40 to 49 years’ old, 50 to 59 years’ old and 60 years and above.

**Statistical analysis:** we performed statistical analysis using Stata 12 software (Statacorp^©^, College Station, Texas, USA). We used Shapiro Wilk test to assess normality of numerical data. We reported mean and standard deviation (SD) for normally distributed data and median and interquartile range (IQR) for skewed data. The appropriate parametric and non-parametric tests were conducted for bi-variate analysis depending on the distribution of the outcome variable and the number of groups compared. Categorical data were reported as percentages and absolute values. Chi squared test was used to compare proportions between racial groups. A p-value of less than 0.05 was interpreted as been statistically significant.

## Results

A total of 21 032 PSA tests were performed on 18 022 participants. We excluded five hundred and forty-one (3%) participants according to study exclusion criteria as shown on Figure 1. After these exclusions, 20 365 PSA results for 17 481 men were included for analysis. Of these, 2876 were for repeat PSA tests requests. We assessed the frequency of PSA repeat per patient. The majority of men (89%) had one PSA test. The rest had multiple PSA tests of which 0.6% had five PSA tests over the 12-month period included in this analysis (data not presented).

**Demographic characteristics of study participants:** the study participants’ demographic characteristics are presented on Table 1. The majority were Black African men (79%), while other race groups comprised 21%. Black African men were younger than men of other racial groups (mean 55 years, SD 13.2 versus mean 61.9 years SD 12.5, p < 0.0005). Most men of other racial groups were 60 years and older (60%). The majority of PSA requests came from clinics compared to hospitals (62% versus 38%) and nurses requested slightly more PSA tests than doctors (51% versus 49%).

**Table 1:** Summary description of study participants

Variable	All participants	Black African	Other
**Number n (%)**	17 481 (100)	13 780 (79)	3701 (21)
**Age (years) mean (SD)**	56.9 (13.3)	55.6 (13.2)	61.9 (12.5)*
**Age (years) categories n (%)**			
< 40	1769 (10)	1597 (12)	172 (5)
40 – 49	3204 (18)	2767 (20)	437 (12)
50 – 59	4761 (28)	3920 (28)	841 (23)
≥ 60	7747 (44)	5496 (40)	2251 (60)
**Type of healthcare facility n (%)**			
Clinic	6686 (38)	9431 (68)	1364 (37)
Hospital	10 795 (62)	4349 (32)	2337 (63)
**Requesting healthcare worker n (%)**			
Doctor	8524 (49)	5828 (42)	2696 (73)
Nurse	8957 (51)	7952 (58)	1005 (27)

* P value < 0.0005

**Prostate specific antigen results by age and race:** prostate specific antigen results were not normally distributed and were therefore summarised using medians and IQR and analysed using non-parametric procedures. The median PSA result for all men was 0.97 µg/L (0.54-2.39 µg/L). Black African men had lower PSA median compare to men of other racial groups (0.95µg/L vs 1.07µg/L, p = 0.038). Most (83%) of the PSA results were between 0.01 and 4.00 µg/L. About one in five men (17%) had PSA result above 4.00 µg/L (Table 2). The analysis of PSA results according to age categories showed that median PSA increased with age (Table 2), ranging from 0.66 µg/L (0.44-1.00 µg/L) in men less than 40 years to 1.68 µg/L (0.72-5.38 µg/L) in men 60 years and older. The median PSA for older Black African men in age categories 3 and 4 (50-59, and 60 years and above) were higher than men of other racial groups (Table 2). Furthermore, as shown in Table 2, fewer Black African men (17%) had PSA levels above 4.00 µg/L compared to men of other racial groups (19%, p = 0.005). As shown in Table 2, the number of men with PSA above age-specific reference range increased with age. More Black African men (33%) aged ≥60 years had PSA level above the age-specific reference range compared to men of other racial groups (26%, p < 0.0005).

**Table 2:** Comparison of PSA results by race

Variable	All participants	Black African	Other	P-value
**PSA (µg/L) median (IQR)**	0.97 (0.54-2.39)	0.95 (0.54-2.27)	1.07 (0.53-2.79)	0.038*
**PSA (µg/L) by age (years) categories median (IQR)**				
< 40	0.66 (0.44-1.00)	0.66 (0.44-0.99)	0.66 (0.40-1.01)	0.711
40 – 49	0.68 (0.46-1.08)	0.68 (0.46-1.08)	0.68 (0.45-1.12)	0.762
50 – 59	0.89 (0.53-1.75)	0.90 (0.54-1.75)	0.85 (0.50-1.74)	0.033*
≥ 60	1.68 (0.72-5.38)	1.79 (0.76-6.03)	1.53 (0.63-4.17)	<0.0005*
**PSA above age specific range n (%)**				
40 – 49	175 (5.5)	155 (5.6)	20 (4.6)	0.381
50 – 59	600 (12.6)	492 (12.6)	108 (12.8)	0.818
≥ 60	2371 (30.6)	1784 (34.5)	587 (26.1)	<0.0005*
**PSA result n (%)**				
≤ 4.00 µg/L	14 467 (83)	11 461 (83)	3006 (81)	
> 4.00 µg/L	3014 (17)	2319 (17)	695 (19)	0.005*

* P-value statistically significant

IQR – interquartile range

**Prostate specific antigen results and prostate biopsy:** only 2% of all men had prostate biopsy. We noted that although Black African had higher PSA median (7.40 µg/L) than men of other racial groups (3.7 µg/L) p = 0.0002, they had significantly fewer biopsies performed: 2% versus 4%, p < 0.0005. Prostate biopsy uptake also differed across all four age categories as depicted in Table 3. More men in age category 4 (≥60 years and older) had biopsies than men of other age categories. When we looked at the age of biopsy and PSA level above specific reference range, we found that more men in age category 4 who had PSA level age-specific reference range had prostate biopsy than men of other age categories. We found that of all men with PSA above 4.00 µg/L, only 8% (n = 245) had prostate biopsy. Across all age groups, prostate biopsy uptake was significantly lower in Black African men than in men of other racial groups except in those age 60 and above where the difference did not reach statistical significance (p = 0.23). For men between the age of 40 and 49 years, Black African men had the lowest prostate biopsy uptake of 0.7%. Furthermore, fewer Black African men (7%) with PSA above 4.00 µg/L had prostate biopsy compared to others (10%).

**Table 3:** Comparison of men who had prostate biopsy by PSA levels, age and race

Variable	All participants	Black African	Other	P-value
**Number n (%)**	423 (2)	276 (2)	147 (4)	0.01*
**Age (years) mean (SD)**	68.0 (8.2)	67.6 (7.9)	68.9 (8.8)	0.142
**Age (years) categories n (%)**				
40 – 49	7 (0.2)	4 (0.1)	3 (0.7)	0.147
50 – 59	52 (1)	33 (0.8)	18 (2)	0.490
≥ 60	363 (5)	238 (4)	125 (5)	0.018*
**PSA (µg/L) median (IQR)**	5.65 (1.29-15.83)	7.40 (1.90-23.2)	3.70(0.80-9.76)	0.0002*
**PSA result n (%)**				
> 4.00 µg/L	245 (8)	175 (7)	70 (10)	0.033*
**PSA above age (years) specific range n (%)**				
40 – 49	3 (0.1)	1 (0.7)	2 (10)	0.002*
50 – 59	33 (0.7)	22 (5)	11 (10)	0.018*
≥ 60	219 (3)	153 (8)	60 (10)	0.227

SD – standard deviation, IQR – interquartile range

* P-value statistically significant

**Prevalence of prostate cancer:** of all men who had prostate biopsy (n = 423), 50% were diagnosed with prostate cancer. Of all men who had a PSA test, 211 had prostate cancer on biopsy which gives prevalence of 1%. The majority of those who had prostate cancer on biopsy were Black African (70%). Among men who had a biopsy, significantly more Black African had prostate cancer compared to men of other racial groups: 54% compared to 43% (p = 0.03). Since NHLS laboratories report PSA results with a cut-off of 4.00 µg/L, we looked at the diagnostic performance of PSA in prostate cancer detection at this cut-off using prostate biopsy as the gold standard. We found that the sensitivity and specificity of PSA at the 4.00µg/L threshold were low. It yielded a sensitivity of 53.3% (95% Confidence Interval: 46.3% to 60.2%) and specificity of 37.1% (95% Confidence Interval: 30.6% to 44.1%).

## Discussion

Using our laboratory data, we have shown that PSA testing is prevalent in our healthcare facilities. The number of PSA tests done differ by age and race. Age specific PSA cut-off levels identified more Black African men with elevated PSA than fixed cut-off of 4.00 µg/L. Biopsy uptake after a PSA test was lower in Black African men, even though they were more likely to be diagnosed with prostate cancer on biopsy. Although there is no screening program for prostate cancer in South Africa, the number of PSA test requests from primary health facilities in our study suggests that opportunistic PSA based screening occur. Studies done in developed countries have shown that even in the absence of a nationwide screening program, PSA based screening does occur [14]. However, there is evidence to suggest that organised PSA based screening is more efficient in reducing prostate cancer mortality than opportunistic testing [21]. It is therefore important for South Africa to ensure that PSA testing in our healthcare facilities become more organised for proper referral, timely diagnosis and treatment to reduce prostate cancer mortality. The finding of high PSA testing in our study is not in keeping with other local studies. A study by Tindall *et al.* done in the rural province of Limpopo and urban city of Pretoria found that of all men referred to the urology clinic, only 3% were due to an elevated PSA [7]. It is important to note that the majority of participants in that study were from the rural areas of Limpopo were PSA based screening is low. On the contrary, our study was done in the urban relatively well-resourced Gauteng province hence the high PSA testing at clinics could be reflecting an increase disease awareness and access to screening facilities amongst Black African men. Additionally, men in rural areas may have higher preferences to traditional medicine than western medicine and therefore not visit healthcare facilities when they have health issues [7].

Other studies conducted in the world and South Africa have described higher PSA levels in Africans across age spectrum [7, 22, 23]. We found an overall lower PSA levels in Black African men compared to others. This could be due to the younger age at PSA testing among Black Africans in our study. The difference in PSA level by race also became notable for men who are 50 years or above. Among men of this age group, PSA levels in Black African men were significantly higher compared to other racial groups from age 50 or above, while in younger men there was no significant difference in PSA levels. Reasons for the high PSA levels amongst African men include high prostate volume, testosterone levels and infections. The small sample size in the younger age categories in our study may explain the homogeneity in PSA levels between racial groups for younger men. It has been found that the type of PSA cut-off used affect clinical interpretation of results. Klinkenberg *et al.* found that fixed PSA cut-offs have higher sensitivity but lower specificity compared to age-specific cut-offs in detecting prostate cancer [24]. Our study found racial disparity when the two types of PSA cut-offs were applied. Less Black African men had a PSA above the 4.00 µg/L cut-off compared to Others. However, when we use the age-specific cut-offs, more Black African men had PSA levels above their age specific cut-offs. This is an important finding as our public sector laboratories still report PSA results with a fixed cut-off of < 4.00 µg/L. Even the few studies conducted in Africa and South Africa have used this fixed cut-off to screen for prostate cancer irrespective of age or race [8, 23]. However, prostate cancer has been found in men with PSA below 4.00 µg/L [25]. In addition, the diagnostic performance of PSA in prostate cancer detection at a cut-off of 4.00 µg/L is low [25]. We also found the sensitivity and specificity of PSA at this cut-off of 4.00 µg/L to be low, 53.3% and 37.1% respectively. Although there is no specific cut-off at which prostate cancer can be excluded. It has been proposed that instead of using PSA level alone to decide on biopsy, other factors such as race, age, digital rectal examination findings, family history of prostate cancer and previous biopsy findings be included in the assessment of individual risk for prostate cancer [25].

Although local guidelines by the South African Prostate Cancer Foundation recommend age-specific cut-offs, there is conflicting evidence on the use of age-specific reference ranges for prostate cancer screening. Some studies have found that PSA age specific cut-offs reduces the number of false negatives in younger men and false positives in older men [26, 27]. In contrast, a study by Catalona *et al.* found that age-specific cut-offs missed 20-60% of cancer in men older than 60 years [28]. In addition, it seems that most assays manufacturers still recommend fixed PSA cut-offs. It is therefore important that we move to establish appropriate total PSA cut-off for prostate cancer screening and diagnosis in African population.

There are no local studies that have looked specifically at the age of PSA testing. Previous studies looking at prostate cancer have shown that Black African men present at an older age and with advanced disease [7, 8, 10]. The younger age of Black African men in our study suggest that this group is becoming more aware of their increased risk of prostate cancer or there is an increased utilisation of PSA in our primary health facilities for screening of prostate cancer. Other studies have found that knowledge about prostate cancer among Black African men is generally low [29, 30]. A local study by Mofolo *et al.* found that more than half of men interviewed at the urology clinic had no knowledge of prostate cancer [29]. Of note is that this study consisted of a significant number of young Black African men ages 34-44 years old [29]. We have also shown that Black African men had lower prostate biopsy uptake but more prostate cancer compared to men of other racial groups. This is in keeping with findings of other local studies [8, 31]. However, we noted in this study that significantly more Black African men had PSA test at primary healthcare facilities which suggest that they were screened. Reasons for low biopsy uptake in this study could be that they did not come back for further investigations, were not referred appropriately or lack of resources to perform biopsies. Ensuring adequate resources for further investigations such as biopsy and educating healthcare workers at primary health facilities about local guidelines for PSA testing and appropriate referral may improve biopsy uptake among Black African men.

Prostate cancer was detected in 1% of all men who had a PSA test. This is in keeping with other studies in the country. In a local study by Heyns *et al.* conducted at a primary health facility in predominantly Black African population living in informal settlements, prostate cancer was detected in 1.4% of all men who were recruited [31]. However, they had low biopsy uptake among Black African in their study due to men not coming back for PSA results and further investigations. Similar to our study, this prevalence is most likely underestimated because of low biopsies. Furthermore, even when they have access to facilities for biopsy and treatment options, biopsy uptake is still low in Black African men [31]. We therefore recommend further larger studies to investigate reasons for low biopsy uptake among Black African men. The strength of our study is that we have presented data on both PSA (n = 20 365) and prostate biopsy in a large sample size from a single province of South Africa. Though, the study had limitations. First, patient files were not reviewed to determine reasons for PSA test requests. Second, not all patients had recorded race information; therefore, those that were clearly identifiable as Black African were categorised accordingly. For the rest we used census data and race imputation program to assign race.

## Conclusion

Prostate specific antigen testing is common in our health care facilities. Many Black African men are tested for PSA levels but have low biopsy uptake in spite of more prostate cancer. We also highlight the need for larger studies to determine appropriate PSA cut-offs for prostate cancer screening in our local population.

### What is known about this topic

- Prostate cancer incidence and mortality rates are highest amongst Black African men in South Africa;
- Black African men are at risk of aggressive form of prostate cancer;
- PSA based screening has been shown to reduce mortality rate in developed countries; however, Black African men present late with advanced disease due to a number of reasons including lack of screening facilities and awareness.

### What this study adds

- This study showed that even in the absence of national screening program, PSA based screening does occur in urban primary health facilities; this highlights the need for education of primary healthcare workers and organised national screening program to ensure proper use of guidelines and patient referral;
- This study also highlights the need for establishing appropriate PSA cut-offs for Black African population for early detection of prostate cancer; in addition, clinicians and the laboratory need to agree on the appropriate cut-offs to ensure proper interpretation of PSA results;
- Furthermore, the findings of this study highlight the need for further larger studies to explore reasons for low biopsy uptake in Black African men.

## Competing interests

The authors declare no competing interests.
